# The effect of meniscal repair on strength deficits 6 months after ACL reconstruction

**DOI:** 10.1007/s00402-020-03347-0

**Published:** 2020-01-29

**Authors:** M. Wenning, A. H. Heitner, M. Mauch, D. Gehring, C. Ramsenthaler, J. Paul

**Affiliations:** 1 Praxisklinik Rennbahn AG, Kriegackerstraße 100, 4132 Muttenz, Basel Switzerland; 2 Department of Orthopedic and Trauma Surgery, Medical Center - University of Freiburg, Faculty of Medicine, University of Freiburg, Hugstetter Str. 55, Freiburg, 79106 Germany; 3 Department of Sport and Sport Science, University of Freiburg, Schwarzwaldstr. 175, Freiburg, 79117 Germany; 4 Cicely Saunders Institute, Department of Palliative Care, Policy and Rehabilitation, King’s College London, London, United Kingdom

**Keywords:** ACL reconstruction, Meniscal repair, Performance testing, Return to sports, Isokinetic strength

## Abstract

**Introduction:**

Ruptures of the anterior cruciate ligament (ACL) can be accompanied by meniscal lesions. Generally, the rehabilitation protocols are altered by meniscal repair. Therefore, the aim of this study was to investigate the effect of meniscal repair on the early recovery of thigh muscle strength in ACL reconstruction (ACLR).

**Materials and methods:**

We performed a matched cohort analysis of *n* = 122 isolated ACLR (CON) compared to *n* = 61 ACLR with meniscal repair (ACLR + MR). The subgroups of meniscal repair consisted of 30 patients who had undergone medial meniscus repairs (MM), 19 lateral meniscus repairs (LM) and 12 repairs of medial and lateral meniscus (BM). Isokinetic strength measurement was performed pre-operatively and 6 months post-surgery to perform a cross-sectional and a longitudinal analysis. All injuries were unilateral, and the outcome measures were compared to the non-affected contralateral leg.

**Results:**

Six months postoperatively overall there is no significant difference between the groups (extension strength MR 82% vs. CON 85% and flexion strength 86% vs. 88%, resp.). Subgroup analysis showed that medial repairs exhibit a comparable leg symmetry while lateral repairs performed worse with leg symmetry being 76% in extension and 81% in flexion strength. Patients undergoing BM repair performed in between lateral and medial repairs (82% extension, 86% flexion).

**Conclusion:**

Generally, meniscal repair in conjunction with ACLR does not significantly alter the recovery of limb symmetry in strength at 6 months postoperatively. Interestingly, medial repairs seem to perform superior to lateral meniscal repair and repair of both menisci. Since the recovery of symmetric strength is a major factor in rehabilitation testing, these results will help to advise surgeons on appropriate rehabilitation protocols and setting realistic goals for the injured athlete.

**Level of evidence:**

III, retrospective cohort study.

## Introduction

When performing anterior cruciate ligament reconstruction (ACLR), one main goal of surgeon and patient is a safe return-to-sport. ACL injuries that occur during pivoting or cutting movements have a relevant risk for associated lesions in the menisci [1]. Recent studies have underlined this co-morbidity in ACL ruptures showing that ACL insufficiency increases the risk and severity of meniscal tears [1, 2]. While return-to-sports in isolated ACLR has been the focus of many publications, little is known about the role that additional meniscal repair may play in this regard [3-6].

Meniscal repair has become a standard procedure accompanying ACLR over the last 2 decades [7, 8]. Generally, it was shown that the outcome of meniscal repair performed at the same time as ACLR has better results than meniscal repair alone [9, 10]. Previous studies have evaluated outcome parameters associated with meniscal repair: The short-term results of meniscal repair in conjunction with ACLR show that patients may have a slightly worse subjective function during the first 6 months [11]. However, the long-term outcome, measured by arthrometric measurements and signs of osteoarthritis, is better whenever the meniscus is preserved [12]. This can be well explained by the additional stability provided by the menisci [13]. Subgroup-analyses suggest that patients requiring medial meniscal repair may have slightly worse long-term outcome in subjective function and higher risk of developing intrameniscal cysts compared to lateral repair [11, 14–16].

When performing meniscal repair, especially in conjunction with ACLR, there is no consensus on the ideal rehabilitation scheme or return-to-play protocol [17, 18]. Furthermore, the rehabilitation schemes differ greatly depending on the surgical technique and the location of the meniscal lesion [17]. Generally, partial weight-bearing and restriction of range-of-motion are frequent during the first postoperative weeks after meniscal repair [17]. This contradicts the current recommendations for rehabilitation following ACLR, in which early weight-bearing and full range-of-motion have been shown to be beneficial [17, 19]. Thus, rehabilitation after meniscal repair may negatively affect the rehabilitation process and subsequently delay return-to-sports [3, 4].

Reducing bilateral strength deficits and normalizing ipsilateral strength balance are important factors for a safe return-to-sport [6]. Several studies have demonstrated imbalances post-ACLR between the operated and the contralateral leg involving knee flexion and extension strength [6]. Strength deficits are the most commonly reported criteria for return-to-play [4]. Furthermore, higher postoperative quadriceps strength is associated with improved return-to-sports [20]. Muscular deficits, however, have been shown to be pronounced within the first 6 months after surgery, while they may persist up to several years [6, 21]. Additionally, persisting strength deficits are associated with a reduced return-to-play rate and worse patient-reported outcomes [3, 22]. It must be stated, however, that the literature on functional measures in the context of return-to-play following meniscal repair is scarce.

### Purpose

Hence, the goal of this study was to analyze the effect of meniscal repair on the strength outcomes 6 months post-ACLR. Second, we performed a subgroup analysis to differentiate the potential outcomes according to the location of the meniscal repair. We hypothesized that the strength deficits would be more pronounced in patients undergoing ACLR with meniscal repair when compared to isolated ACLR.

## Methods

This is a retrospective analysis of our prospectively collected data of patients treated with ACLR between 12/2015 and 04/2017. All procedures were performed at our orthopedic hospital by a total of five different surgeons following the same standardized procedure. This study was approved by the local ethics committee (EKNZ 2017-01825) and performed according to the Declaration of Helsinki in its current form.

### Patients

**Fig. 1:**
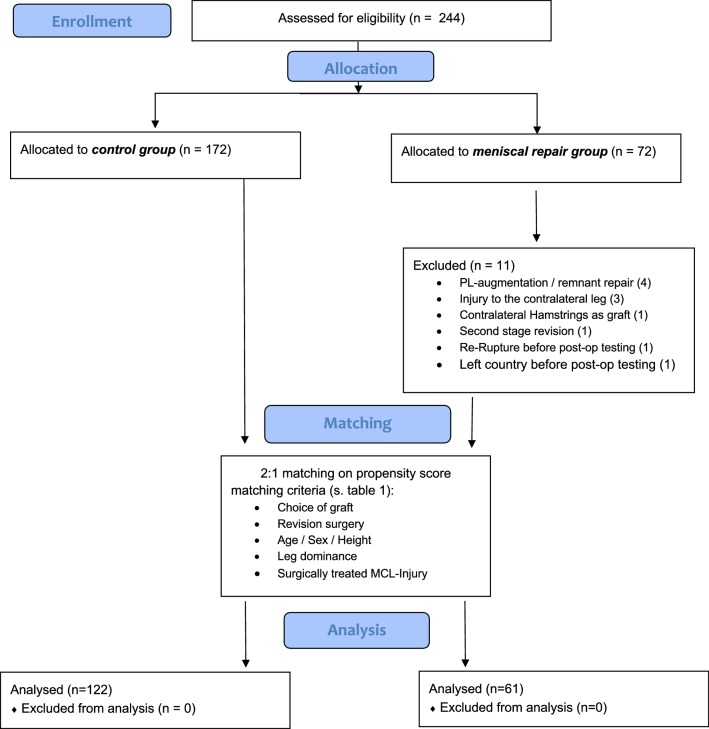
Flowcart showing patient enrollment and allocation

We screened the medical records of 221 patients that were scheduled for ACL reconstruction. Inclusion criteria for the meniscal repair group were unilateral ACLR with meniscal repair in the same session. The matched control group had undergone unilateral ACLR without meniscal repair. Patients undergoing partial meniscectomy were also included in the control group. Exclusion criteria for both groups were second-stage revision, additional cartilage procedures (microfracturing, matrix associated chondrogenesis) or osteotomies performed on either leg. Furthermore, we excluded all patients that had suffered relevant injuries to either leg like contralateral ACL ruptures and previous tendon or muscular injuries of the lower limbs. Associated treatments like partial meniscectomy or superficial chondroplasty with no effect on the postoperative proceedings were not specifically recorded. Figure 1 summarizes patient recruitment in a flowchart.

A total of 61 patients met our inclusion criteria for the meniscal repair group (ACLR + MR). For the control group, *n* = 122 was chosen as a propensity-matched control group (ACLR) which was matched for the choice of graft, age decade, sex, height and revision ACLR. This matching resulted in two cohorts with a balanced distribution of covariates (Table 1).

**Table 1:** Composition of the two matched cohorts

Variable	Meniscal repair group (*n* = 61)		Matched control group (*n* = 122)	
	*n*	%	*n*	%
Gender				
Men	42	68.9	84	68.9
Women	19	31.1	38	31.1
Age at surgery [in years, mean (SD)]	27.9 (10.9)		28.9 (10.5)	
10–19	16	26.2	26	21.3
20–29	25	41.0	46	37.7
30–39	8	13.1	22	18.0
40–49	10	16.4	19	15.6
50–59	2	3.3	6	4.9
Height [in m, mean (SD)]	1.76 (7.9)		1.75 (8.1)	
Weight [in kg, mean (SD)]	75.4 (10.6)		75.8 (15.3)	
BMI [median (IQR)]	23.9 (22.2–26.3)		24.1 (22.1–26.2)	
Graft choice				
ST	44	72.1	95	78.0
STG	7	11.5	9	7.4
Allograft	5	8.2	16	13.1
BPTB	5	8.2	2	1.6
First-time revision ACLR	9	14.8	16	13.1
Injured vs. dominant leg				
Injured dominant leg	34	55.7	68	55.7
Injured non-dominant leg	27	44.3	54	44.3

*ACLR* anterior cruciate ligament reconstruction, *SD* standard deviation, *IQR* interquartile range, *ST* semitendinosus tendon, *STG* semi-tendinosus and gracilis tendon, *BPTB* bone–patellar tendon–bone

### Subgroups

The meniscal repair group was further divided according to the location of meniscal repair, with *n* = 30 medial meniscal repair (MM), *n* = 19 lateral meniscal repair (LM) and *n* = 12 meniscal repair in both compartments (BM) (see below).

Of the 30 patients undergoing medial meniscal repair, 24 of these lesions were in the posterior horn, two bucket-handle lesions and four lesions were primarily in the pars intermedia. Of the 19 patients that had a repair of the lateral meniscus (LM), 14 were in the posterior part, 3 in the pars intermedia, 1 in the anterior horn and 1 meniscal root repair. Of the 12 patients that were repaired on the lateral and medial meniscus, 7 had both lesions in the posterior horn, 3 were not specified separately and 2 underwent repair of a lateral root tear combined with a medial posterior horn repair.

### Surgical technique

For ACLR, we used a proximal extra-cortical fixation (Endobutton CL Ultra, Smith&Nephew, London, UK) and a tibial hybrid fixation using a bioresorbable interference screw and additionally extra-cortical fixation with the femoral tunnel drilled via the anteromedial portal.

The meniscal repairs were performed as follows: posterior horn repairs were performed using an all-inside technique (FastFix, Smith&Nephew, London, UK), the anterior lesion was repaired using an outside-in technique and the three root tears were arthroscopically reconstructed via an additional transtibial drilling with extracortical button fixation (EndoButton CL Ultra, Smith&Nephew, London, UK). Repairs of bucket-handle lesions were performed combining all-inside techniques (FastFix, Smith&Nephew, London, UK), and inside-out techniques using non-resorbable sutures (PDS 2-0).

### Rehabilitation scheme

The postoperative rehabilitation scheme was highly standardized for all patients where isolated ACLR was performed; in these patients, immediate full weight-bearing was allowed. The knee flexion angle was limited at 90° for 2 weeks and no knee-orthosis was used.

In those patients undergoing an isolated medial meniscus repair, the passive flexion limit was kept at 90° for 6 weeks; however, immediate full weight-bearing was allowed while keeping the leg in full extension and wearing external bracing during mobilization. Only the patients after a bucket-handle repair had partial weight-bearing (15 kg) for 3 weeks.

In lateral meniscal repair, flexion angle was limited according to the location and severity of the tear; partial weight-bearing was recommended for 3 weeks with a flexion limit of 60° for 3 weeks and 90° for another 3 weeks. In the three cases of meniscal root fixation, no weight-bearing was allowed for 6 weeks with passive flexion angles of 60–90° during that time.

Generally, after the first 6 weeks, the progression within the individual rehabilitation scheme was criterion based [23]. One key factor in allowing a progressive weight-bearing was the focused activation of the quadriceps muscle to allow active anterior–posterior stabilization. Furthermore, in cases of postoperative flexion limit, a gradual increase using continuous passive motion machines was recommended until reaching 90° of knee flexion. Before restarting running activity, an adequate stabilization of a single leg stance was required. Running activity was initially supported using an anti-gravity treadmill; generally, most patients returned to running about 4–5 months postoperatively.

### Strength measurements

The functional testing was performed preoperatively and on average 26 weeks post-surgery. However, those patients undergoing surgery within the first few days after the accident, suffering from meniscal impingement or other reasons of limited preoperative ROM like bucket-handle lesions, did not perform preoperative isokinetic strength measurements. The modalities of the strength measurements used in this study are as previously described and in accordance with the current recommendations in the literature [24]. For all strength measurements, we used an isokinetic dynamometer (Humac Norm, CSMi, Stoughton, USA).

Concentric peak torque in flexion and extension was measured as the average of five repetitions at a 60°/sec dynamometer speed. Prior to strength assessments, three submaximal trials were applied for familiarization. Isokinetic testing was completed with maximal effort and verbal encouragement in concentric–concentric mode. During strength assessment, patients were sitting upright, upper body fixed, hands at the grips, while the leg was tightly fixed at the thigh with the lever arm positioned at two-thirds of the lower leg.

### Statistical analysis

Missing data were explored according to their pattern and cause [25]. The mechanism behind missing data followed a missing completely at random pattern. In logistic regression analyses, predictors for missingness were determined based on demographic and clinical characteristics and those predictors found significant were used to estimate missing data in multiple imputations. All statistical analyses were run as complete case analyses and then contrasted in a sensitivity analysis with multiple imputations of missing data [25].

Prior to statistical analyses, assumptions for independent and dependent samples, Student’s *t* tests as well as repeated measures analysis of variance to compare outcomes in operated and non-affected limbs in each group and to compare outcomes over time between groups were tested. The presence of normal distributions and the amount of outliers in outcomes were checked using data exploration techniques. To remedy problems with assumptions, outlying observations were shifted to the respective lower and upper ends of 1.5 times the interquartile range to truncate their influence on the data [26].

Two main analyses were run: one to compare outcomes between the ACLR and the matched control group over time, a repeated measures analysis of variance (*rmANOVA*) was conducted with a main factor for group (ACLR vs. matched control group) and two-level factor time (pre/post) for each outcome, respectively. Mauchly’s test of sphericity was used to check assumptions with the Greenhouse–Geisser correction employed if the assumption of sphericity was violated. The level of significance was defined at *p* < 0.05. In addition to statistical significance, effect sizes eta squared (*η*^2^) and percentage change (observed difference to the total amount of difference over time) were calculated for the pairwise comparisons of the repeated measure factor time. Effect sizes were interpreted following Cohen [27] as small: 0.01, medium: 0.06, and large: 0.12. A second analysis was run comparing strength outcomes between operated and uninjured limbs in each ACLR repair group (medial, lateral, both medial and lateral and no repair group) using dependent sample Student’s *t* tests. Due to the high number of statistical tests, statistically significant p values were Bonferroni corrected to a p value of *p* < 0.002.

Due to the retrospective nature of the study, an a priori sample size calculation was not possible. However, we calculated the maximum effect sizes that could be obtained given the data that were available to estimate type II error. For the first analysis using rm-ANOVA, a power of 0.8 and an alpha-error of 0.05 at a medium correlation of 0.5 between the repeated measurements would yield effect sizes of partial *η*^2^ = 0.02 (*f* = 0.13). For the second analysis, we performed a sample size calculation for a matched pair t test assuming an alpha-error of 0.05, a power of 0.8 and an effect size of 0.5 which lead to a minimum group size of *n* = 34.

Statistical analysis was conducted using the Statistical Package for the Social Sciences (SPSS) Version 24 [28] and “R” [29], sample size and sensitivity analyses were conducted using G*Power v. 3.9.1.4. Graphical display was performed using Veusz (Veusz v. 3.0.1).

## Results

The amount of missing data in the pre-operative assessment was higher than at the 26 weeks post-surgery measurement time point. On average, 33% of data were missing pre-surgery. This dropped to 14% at the post-operative time point.

### Longitudinal analysis

**Fig. 2:**
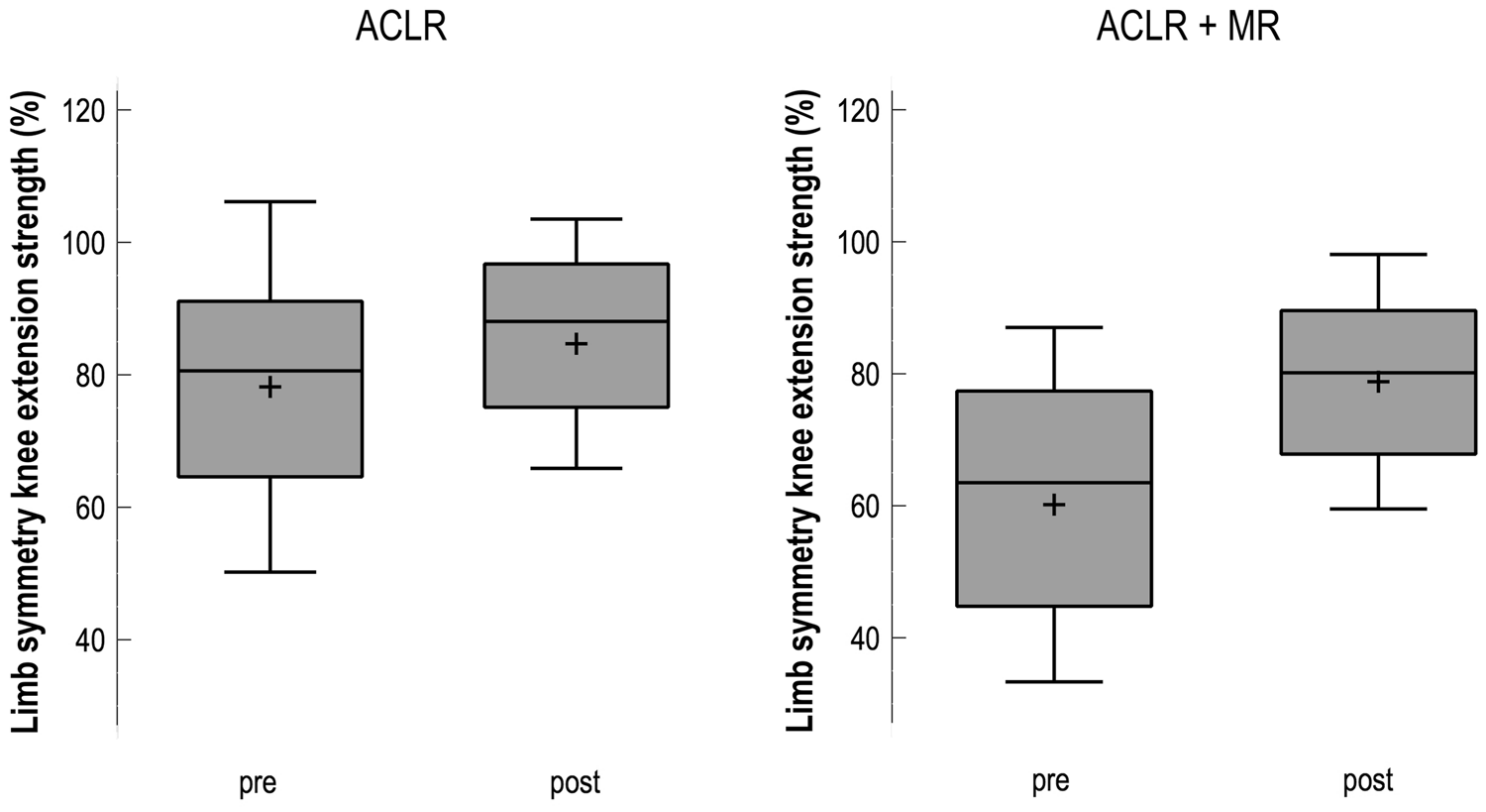
Box plots (median, mean and SD) showing limb symmetry (%) in knee extension strength preoperatively (pre) and 6 months postoperatively (post). *ACLR* anterior cruciate ligament reconstruction (*n* = 122), *ACLR + MR* anterior cruciate ligament reconstruction with additional meniscal repair (*n* = 61)

**Fig. 3:**
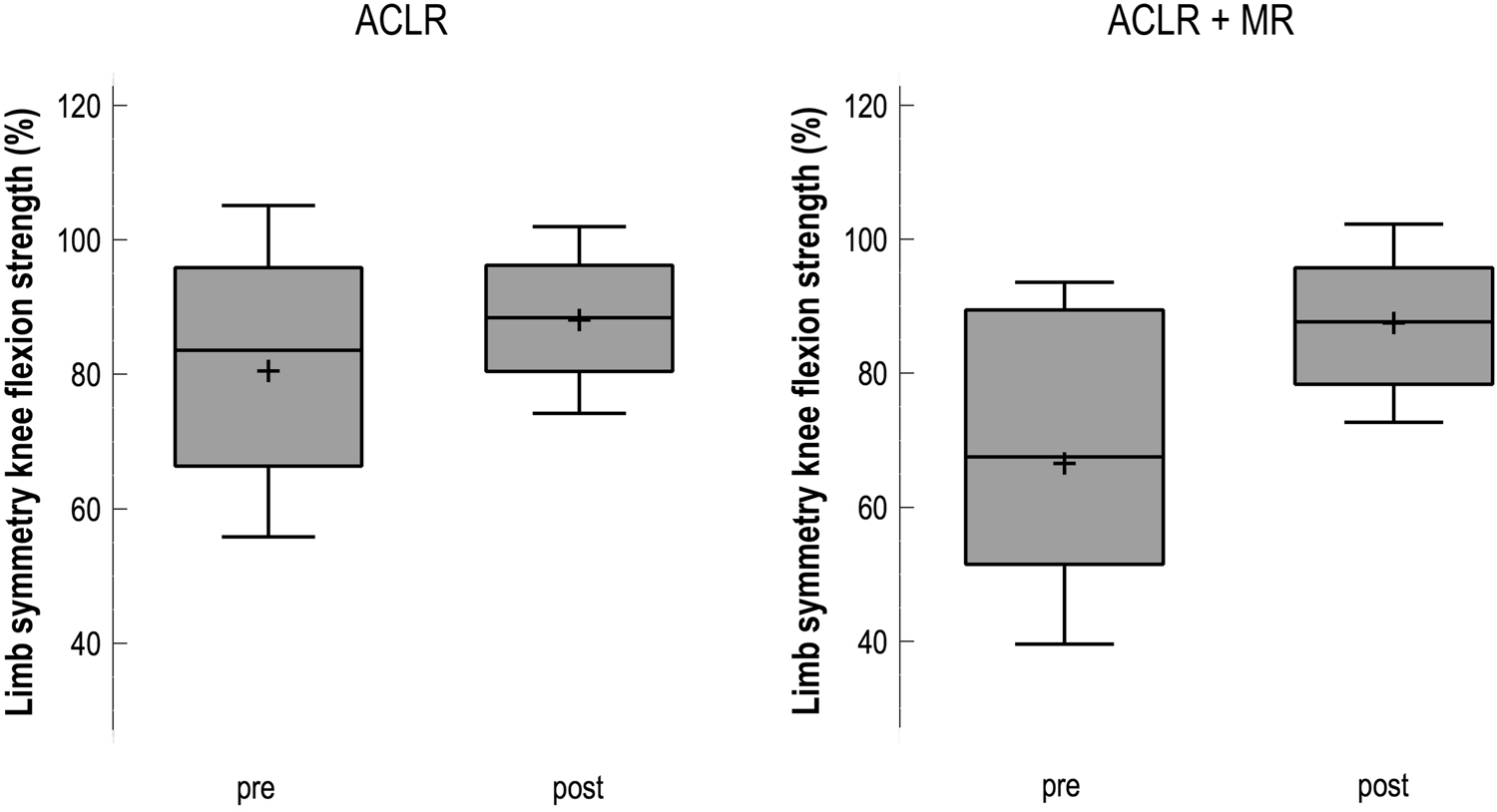
Box plots (median, mean and SD) showing limb symmetry (%) in knee flexion strength preoperatively (pre) and 6 months postoperatively (post). *ACLR* anterior cruciate ligament reconstruction (*n* = 122), *ACLR + MR* anterior cruciate ligament reconstruction with additional meniscal repair (*n* = 61)

Table 2 presents the calculations for the between-group analyses and the effect size of changes from pre- to post-surgery. Over the course of rehabilitation, all absolute strength values of the operated limb improved significantly (*p* < 0.05). The meniscal repair group had a higher preoperative deficit in knee extension strength when compared to controls (*p* = 0.07). Thus, the improvement during rehabilitation was greater in this group than in controls (19% vs. 5%). For the control group, also the non-affected limb showed significant improvements over time in extension (*p* = 0.04) and flexion (*p* = 0.03) strength, whereas the meniscal repair group’s healthy leg did not change. The limb symmetry for extension strength improved significantly in both groups (CON pre 80% to post 85% and MEN pre 63% to post 82%, see Fig. 2). The absolute strength as well as the leg symmetry for knee flexion strength (see Fig. 3) showed comparable values for meniscal repair and CON. There were no significant group x time interactions as shown in Table 2. Also, H/Q-ratio did not change over time and effect sizes were negligible.

**Table 2:** Knee extension strength, knee flexion strength, corresponding limb symmetry and hamstring quadriceps ratios for the meniscal repair group vs. matched control group pre and 6 months post-surgery

	Meniscal repair group (*n* = 61)			Matched control group (*n* = 122)			*rm*ANOVA
	Pre (mean ± SD)	Post (mean ± SD)	*η*^2^	Pre (mean ± SD)	Post (mean ± SD)	*η*^2^	
Extension strength							
OP (Nm)	**88.12 ± 46.3**	117.8 ± 44.1*	0.06	**101.5 ± 44.2**	121.1 ± 45.4*	0.21^a^	*p* = 0.102 *F *(1. 181) = 2.703
NOP (Nm)	139.3 ± 43.3	142.9 ± 41.0	0.01	**126.8 ± 43.0**	143.1 ± 44.8*	0.06	*p* = 0.214 *F *(1. 181) = 1.554
Sym (%)	**63.2 ± 22.2**	82.4 ± 16.9*	0.12	**80.0 ± 23.8**	84.6 ± 17.7*	0.12	*p* = 0.095 *F *(1. 181) = 2.819
Flexion strength							
OP (Nm)	**73.2 ± 27.1**	84.5 ± 23.6*	0.09	**70.2 ± 32.5**	86.5 ± 29.6*	0.15^a^	*p* = 0.408 *F *(1. 181) = 0.688
NOP (Nm)	97.4 ± 27.7	98.4 ± 26.8	0.01	**89.4 ± 27.2**	98.0 ± 29.0*	0.05	*p* = 0.205 *F *(1. 181) = 1.619
Sym (%)	**75.2 ± 23.7**	85.9 ± 12.7*	0.16^a^	**78.5 ± 24.2**	88.3 ± 13.2*	0.21^a^	*p* = 0.825 *F *(1. 181) = 0.09
H/Q ratio							
OP	75.3 ± 16.6	79.4 ± 20.3	0.02	75.4 ± 20.3	74.30 ± 17.5	0.01	*p* = 0.228 *F *(1. 181) = 1.463
NOP	69.7 ± 11.3	69.5 ± 13.1	0.01	71.4 ± 12.0	69.7 ± 9.9	0.01	*p* = 0.548 *F* (1. 181) = 0.362

Bold values indicate significant difference

*OP*  operated leg, *NOP*  non-affected leg, *Sym*  limb symmetry, *Nm*  Newton meter, *SD* standard deviation

^a^High effect size (*η*^*2*^ > 0.12)

*Significant changes pre/post within group and limb as pairwise comparisons with *p* < 0.05 in repeated-measures ANOVA

### Cross-sectional analysis and meniscal repair subgroups

At 6 months post-surgery, the cross-sectional analysis of the subgroups for meniscal repair revealed several differences between the different locations of meniscal repair as shown in Table 3. Generally, all groups still show a relevant side-to-side deficit, where the operated leg achieves lower values for extension and flexion strength.

**Table 3:** Comparison of knee extension strength, knee flexion strength and H/Q-ratio at 6 months post-surgery between the operated and the non-affected leg in each subgroup

		Extension strength	Limb symmetry extension strength	Flexion strength	Limb symmetry flexion strength	H/Q ratio
		Mean ± SD (Nm)	Mean ± SD (%)	Mean ± SD (Nm)	Mean ± SD (%)	Mean ± SD
CON	OP	**121.1 ± 45.1**	84.6 ± 18	**86.5 ± 29.0**	88.3 ± 13	**74.3 ± 17.5**
*n* = 122	NOP	143.1 ± 44.8*		98.0 ± 27.3*		69.7 ± 9.9*
MM	OP	**121.4 ± 40.9**	87.2 ± 16	**86.4 ± 21.1**	89.0 ± 12	**78.2 ± 15.2**
*n* = 30	NOP	139.3 ± 37.1*		97.1 ± 26.3*		68.3 ± 10.1*
LM	OP	**113.3 ± 41.3**	75.7 ± 13	**82.5 ± 25.5**	81.1 ± 12	77.6 ± 14.3
*n* = 19	NOP	149.7 ± 38.8*		101.7 ± 26.4*		68.8 ± 9.3
BM	OP	116.0 ± 58.0	82.3 ± 24	83.0 ± 27.9	86.0 ± 15	85.5 ± 35.3
*n* = 12	NOP	141.4 ± 53.9		96.5 ± 30.4		73.8 ± 22.2

Bold values indicate significant difference

*MM* medial meniscal repair, *LM* lateral meniscal repair, BM medial and lateral meniscal repair, *CON* isolated ACLR, *OP* operated limb, *NOP* non-affected limb, *Nm* Newton meter, *SD* standard deviation

*Significant (*p* < 0.002) differences between the operated and non-affected limb within the respective group

Overall MM repair showed higher limb symmetry in flexion (89%) and extension (82%) strength when compared to LM (81% and 76% resp.) or BM (86% and 82% resp.), but significance was not reached between the subgroups. Also, the values of MM were comparable to the values observed for CON (88% and 85%) and no significant differences between the groups were found. Lateral meniscal repair showed the overall lowest values for limb symmetry while the absolute strength values for the non-affected limb were the highest. Across all groups, the H/Q ratio was higher for the operated limb when compared to the non-affected limb.

## Discussion

The most important finding of this study was that the effect of meniscal repair performed in conjunction with ACLR does not necessarily alter isokinetic strength performance at 6 months postoperatively. According to the location of the meniscal lesion, it seems that lateral repair performs worse than medial meniscal repair. These results may be used to advise patients undergoing ACLR striving to return to play as soon yet as safe as possible [30].

Preserving the meniscus in ACLR should be sought for whenever possible [31]. It improves subjective outcomes, objective knee stability, shows lower re-operation rates and prevents the progression of osteoarthritis in the long term [1, 9, 14, 32, 33]. At the same time, current research is underlining that early ACLR improves the outcome after meniscal repair in conjunction with ACLR while protecting the knee from secondary injury like chondral lesions and aggravated meniscal lesions [1, 2, 34]. While osseous factors like tunnel positioning and tibial slope are established important factors for ACL graft failure, the role of periarticular structures, meniscal kinematics and strength deficits following ACLR is still a major focus of current research [6, 35–39].

In addition to these beneficial long-term effects, our study revealed that short-term function is only slightly lower in some patients, but overall not significantly altered by meniscal repair. This evidence will encourage the ambitious athlete, showing that recovery of thigh muscle strength seems not to be significantly delayed by meniscal repair [30, 40]. This is important since the recovery of strength balance is one major factor in clearing athletes for a safe return-to-competition [6, 41].

### Longitudinal analysis

Those patients undergoing meniscal repair showed inferior strength and leg symmetry at the time of surgery when compared to controls. This is in line with the literature where patients undergoing meniscal intervention had shown inferior preoperative function and performance [11, 14, 42, 43]. Possibly, the greater amount of damaged tissue causes a more severe arthrogenic inhibition of the periarticular muscles [44, 45]. This inhibition as well as local factors such as pain, swelling and inflammation may disappear once the meniscal integrity is restored [17]. Consequently, the meniscal repair group did not show a pronounced deficit in extension strength at the postoperative testing. However, this somewhat contradicts earlier findings, where it was shown that preoperative quadriceps strength correlates to postoperative strength recovery [22, 42]. Furthermore, it was suggested that the partial weight-bearing during rehabilitation of meniscal repairs reduces quadriceps strength [11, 17, 46]. However, our results indicate that it is possible for the athlete to regain quadriceps strength after ACLR + MR within the same time period as isolated ACLR. Interestingly, limb symmetry in knee flexion strength was generally higher than in quadriceps strength and effect sizes were stronger. Despite harvesting hamstring tendons in the majority of the patients (72–78%) the recovery of knee flexion strength was more symmetric at 6 months postoperatively compared to knee extension strength. This may be due to the finding that arthrogenic muscle inhibition primarily affects the quadriceps muscle which causes a prolonged strength deficit in knee extension compared to flexor strength [45, 47]. All other established factors affecting quadriceps strength after ACLR like age and gender were equally distributed across the groups.

### Cross-sectional analysis of subgroups

The importance of achieving an adequate limb symmetry in strength before returning to the field is well accepted [6, 24, 30]. Return-to-play criteria mostly require a recovery of > 85% of the healthy limb’s strength [3, 6, 20]. In our subgroup analysis, we were able to show that only medial meniscal repair (MM 87.2%) fulfilled this criterion, while repairs of the lateral meniscus (LM 75.7%) and repair of both menisci (BM 82.3%) still exhibited a greater deficit. Contrarily, a recent analysis revealed that medial as well as lateral repair is associated with reduced quadriceps strength at 6 months postoperatively, while knee flexion strength was not significantly reduced [46]. Cristiani et al. attributed this to the early restriction in range of motion and partial weight-bearing [46]. However, scientific evidence on the effect of these limitations is scarce.

Since strength asymmetry has been linked to subjective knee function post ACLR [48], the strength deficit observed in our study may explain why lateral meniscal repairs also exhibit worse subjective function at 6 months post-surgery [11]. In previous studies, a comparable subjective function after meniscal repair was achieved as late as 1 and 2 years post-surgery [11, 14]. A limb symmetry below the cut-off value of 85% was found in the LM and BM groups, which must be considered clinically relevant [6]. Thus, lateral meniscal repair may require more time before successfully passing this return-to-play criterion. It may be suggested that this is due to the initial restrictions (range of motion and weight-bearing), which may have a persisting negative effect on strength recovery. However, the results of this subgroup analysis are of explorative character and they do not allow for a causative interpretation.

Furthermore, it needs to be stated, that the interindividual variability was relatively high; hence, none of the differences between the subgroups reached statistical significance when adapted for multiple testing. This underlines the observation that the individual recovery from ACLR varies greatly [30]. In line with current publications, this supports a criterion-based rehabilitation over an isolated time-based approach [30, 49, 50].

Limitations of the study include the relatively high rate of missing data from pre-operative isokinetic analysis, which is owed to the fact that patients with effusion, pain or unstable meniscal lesions were excluded from preoperative functional analysis. Furthermore, sample size calculation suggested that a type two error might be present in the subgroup analysis and requires a careful interpretation especially of the results in LM and BM.

## Conclusions

This study revealed that meniscal rupture and repair, when performed in conjunction with ACLR, has no significant effect on isokinetic strength outcome 6 months after surgery. Despite the more conservative rehabilitation in the first postoperative weeks, patients seem to recover their strength as quickly as 6 months postoperatively. However, the location of the meniscal lesion seems to influence the recovery of strength with lateral menisci performing worse than medial meniscal repairs.

## Abbreviations

ACL: Anterior cruciate ligament
ACLR: Anterior cruciate ligament reconstruction
MR: Meniscal repair
CON: Control group (isolated ACLR)
MM: Medial meniscal repair
LM: Lateral meniscal repair
BM: Both menisci repaired
OP: Operated limb
NOP: Non-operated limb
